# Synergetic regulation of translational reading-frame switch by ligand-responsive RNAs in mammalian cells

**DOI:** 10.1093/nar/gku1233

**Published:** 2014-11-20

**Authors:** Hsiu-Ting Hsu, Ya-Hui Lin, Kung-Yao Chang

**Affiliations:** Institute of Biochemistry, National Chung-Hsing University, 250 Kuo-Kung Road, Taichung 402, Taiwan

## Abstract

Distinct translational initiation mechanisms between prokaryotes and eukaryotes limit the exploitation of prokaryotic riboswitch repertoire for regulatory RNA circuit construction in mammalian application. Here, we explored programmed ribosomal frameshifting (PRF) as the regulatory gene expression platform for engineered ligand-responsive RNA devices in higher eukaryotes. Regulation was enabled by designed ligand-dependent conformational rearrangements of the two cis-acting RNA motifs of opposite activity in -1 PRF. Particularly, RNA elements responsive to trans-acting ligands can be tailored to modify co-translational RNA refolding dynamics of a hairpin upstream of frameshifting site to achieve reversible and adjustable -1 PRF attenuating activity. Combined with a ligand-responsive stimulator, synthetic RNA devices for synergetic translational-elongation control of gene expression can be constructed. Due to the similarity between co-transcriptional RNA hairpin folding and co-translational RNA hairpin refolding, the RNA-responsive ligand repertoire provided in prokaryotic systems thus becomes accessible to gene-regulatory circuit construction for synthetic biology application in mammalian cells.

## INTRODUCTION

The dynamic transition of alternative RNA conformations has been exploited by nature to regulate RNA-dependent cellular functions. This is achieved through regulatory formation of specific RNA structures embedded in distinct RNA-mediated functional platforms (1). Riboswitches, RNA elements responsive to metabolites, have been widely used to regulate transcription termination or translation initiation for tuning specific gene expressions in response to nutrient variations in prokaryotes (2,3). Interestingly, only thiamine pyrophosphate (TPP) riboswitch has been demonstrated in eukaryotic systems (fungi and plants) (4); as yet, no natural riboswitch has been characterized in mammalian systems (5). Extensive efforts have been devoted to looking for chemical scaffolds capable of triggering riboswitch-mediated gene expression in both prokaryotic and eukaryotic systems. Such research is aimed at discovering biomedical and synthetic biology applications (6,7). There have been few successful examples of engineered mammalian riboswitches (8,9).

Limited success in mammalian-riboswitch applications may relate to mammalian systems using different mechanisms from those of prokaryotes to terminate transcription and initiate translation. The discovery of alternative splicing regulation by TPP riboswitches in fungi and plants (4) suggests that other RNA-mediated gene-expression platforms may provide a better framework for constructing successful eukaryotic riboswitches. Recently, engineered metabolite-responsive RNA pseudo-knots of prokaryotic origin were shown to possess ligand-specific -1 programmed ribosomal frameshifting (-1 PRF) stimulation activity in reticulocyte lysate (10,11). The -1 PRF causes an elongating ribosome to shift a single nucleotide in the 5′-direction of mRNA, leading to a -1 reading-frame switch during decoding. Efficient -1 PRF requires a slippery sequence (where frameshifting occurs) and an optimally placed downstream stimulator structure (usually a pseudo-knot) (12). Indeed, translational frameshifts have been found to serve as nutrient sensors (13). However, while the success of converting a ligand-responsive pseudo-knot into a ligand-dependent -1 PRF stimulator suggests that translational reading-frame switch regulation holds promise as an expression platform for engineering mammalian riboswitches, its general application is hampered by the difficulty in finding specific ligand-responsive -1 PRF pseudo-knots (10,11).

In this study we take advantage of the newly found upstream -1 PRF attenuator hairpin (14) to propose an alternative approach for constructing ligand-responsive -1 PRF regulatory element. Similarity between the folding of an RNA polymerase transcribing RNA hairpin and the refolding of a ribosome unwound RNA hairpin suggests a robust way to transforming identified ligand-responsive RNA elements in prokaryotic transcriptional termination regulation into translational elongation control modules by using the elements to regulating upstream -1 PRF attenuator hairpin refolding. Accordingly, this novel approach makes the prokaryotic riboswitch repertoire available for mammalian application.

## MATERIALS AND METHODS

### Plasmids and construction of reporters

Three different -1 PRF reporters were used in this study. The p2luc recoding reporter (15) was obtained from Professor John Atkins at the University of Utah. To facilitate -1 PRF activity analysis *in vitro*, a premature -1 frame stop codon was introduced 33 nucleotides downstream of the *BamH*I site of p2luc, leading to the generation of a shortened -1 frame product during translation in reticulocyte lysate.

To generate a Venus-based reporter suitable for -1 PRF activity analysis in 293T cell, gene fragment encoding N-Venus (residues 1–174) or C-Venus (residues 175–240) was amplified using pNPY-Venus-N1 (provided by Professor A. Miyawaki at RIKEN) as the template. Primers F1 and R1 were used to amplify N-Venus to create an extended C-terminal linker with its terminal 29 nucleotides sequences (underlined) overlapped with nucleotide sequences of the extended N-terminal linker of the C-Venus fragment amplified by primers F2 and R2. The underlined complementary region in R1 and F2 primers both contain *Sal*I and *BamH*I restriction sequences (typed boldly) that can provide the insertion site for frameshifting element cloning in the fluorescent -1 PRF reporter. The two amplified gene fragments were then fused by the polymerase chain reaction (PCR)-based ligation approach (16) using F1 and R2 as primers to generate a composite gene fragment with a cloning sites embedded linker sequence connecting nucleotide sequences corresponding to residues 174 and 175 of the original Venus open reading frame (ORF). The linker inserted Venus fragments were then used to replace the original Venus in pNPY-Venus-N1 to generate a vector pNinsertC-Venus.

F1:5′-CCCAAGCTTAATACGACTCACTATAGGGAGACCCAATCGCCACCATGGTGAGCAAGG-3′
R1:5′-GAAGTTGAA**GGATCC**GGTACC**GTCGAC**ATGTCCTCGATGTTGTGGCGGATCTTGAAG-3′
F2:5′-AT**GTCGAC**GGTACC**GGATCC**TTCAACTTCCCTGAGGGCGGCGTGCAGCTCGCC-3′
R2: 5′-TGATCTAGAGTCGCGGCCGCT-3′

### Recombinant DNAs and mutagenesis

-1 PRF elements containing different combination of pseudo-knot stimulators and upstream attenuator sequences were chemically synthesized and purchased from Mission Biotech, Taiwan. Longer elements were constructed by assembling different pieces of chemically synthesized DNA oligo-nucleotides with partially overlapping sequences via the PCR-based ligation approach (16). Forward and reverse DNA primers, respectively, carrying *Sal*I and *BamH*I restriction sites and appropriately designed annealing sequences, were used for PCR amplification of the cDNAs encoding -1 PRF elements of interest. The amplified inserts of interest were then cloned into the *Sal*I/*BamH*I sites of different -1 PRF reporters as needed. Cloning was conducted using standard procedures and the resultant recombinant vectors were transformed into the DH5α strain of *Escherichia coli* cells for maintenance and selection by ampicillin or kanamycin. Mutagenesis was constructed using the quick-change mutagenesis kit from Stratagene according to the manufacturer's instructions.

The genes encoding the CAT domain (residues 1–67) of *Bacillus subtilis* GlcT anti-terminator protein were amplified by PCR using bacterial chromosome extracted from *B. subtilis* DB2 (a gift from Professor B.Y. Chang at NCHU) with primers (F3 and R3) containing restriction enzymes *BamH*I and *EcoR*I recognition sequences (underlined). The amplified gene fragments as well as the pGEX-4T1 expression vector (GE Lifescience Technology) were then treated with the two restriction enzymes, purified and ligated by T4 DNA ligase (Takara) to obtain a recombinant GST-GlcTRBD expression vector. The identities of all cloned and mutated genes were confirmed by DNA sequencing analyses.

F3:5′-CGGGATCCATGACAAAGGAGCTGAGGATCGTGAATGG-3′
R3:5′-GGAATTCTTATTGTTCCTTCTCGTCTTTTAAAATGAACATTTTATGCTAGCC-3′.

### RNA synthesis and purification

Synthetic ribonucleic-antiterminator (RAT) RNAs used in this study were transcribed by T7 RNA polymerase with designed DNA templates using *in vitro* transcription method (17). After being purified by 20% denaturing polyacrylamide gel electrophoresis (PAGE) in the presence of 8 M urea, the gels of bands containing desired RNA sequences were cut out and electro-eluted using a BIOTRAP device (Schleicher & Schuell). The eluted RNAs were then ethanol precipitated and recovered by centrifugation. The concentration of RNA was determined by ultraviolet absorbance at 260 nm.

### Expression and purification of fusion proteins

*E. coli.* BL21 (DE3) cell was used as the host for the expression of GST-tagged GlcT proteins. The transformed bacteria were grown with LB media in the presence of 100 μg/ml ampicillin to an A^600^ value of 0.6 at 37°C. Expression of proteins was then induced by the addition of 0.1 mM isopropyl-1-thio-β-D-galactopyranoside and cells grown for a further 3–5 h. Cells were then collected by centrifugation and suspended in phosphate buffered saline (PBS) buffer (10 mM Na_2_HPO_4_, 1.8 mM KH_2_PO_4_, 140 mM NaCl, 2.7 mM KCl, 0.2 mM phenylmethylsulfonyl fluoride [PMSF], pH 7.3) for storage at −20°C. To purify expressed proteins, the frozen cell pellets containing expressed GST-fusion proteins were thawed and sonicated in the presence of 1 mM PMSF. The cell lysates were centrifuged and the clarified lysates were then loaded into a pre-packed glutathione-agarose bead column (GE Lifescience Technology) equilibrated with 1× PBS buffer and washed with 10 column volumes of PBS buffer. The GST-tagged proteins were eluted with 1 column volume of elution buffer (50 mM Tris-HCl and 10 mM reduced glutathione at pH of 8.0). The fractions containing eluted fusion proteins were checked by sodium dodecyl sulfate-PAGE (SDS-PAGE) and Coomassie Brilliant Blue staining, pooled and dialyzed against PBS buffer containing 50% glycerol overnight at 4°C, and stored as separated aliquots at −20°C. Protein purity was examined by SDS*-*PAGE, while the protein concentration was measured using a Bradford assay (BioRad).

### Radioactivity-based *in vitro* -1 PRF assay

Capped reporter mRNAs were prepared using a mMESSAGE mMACHINE high-yield capped RNA transcription kit (Ambion) by following manufacturer's instructions. Reticulocyte lysate (Ambion) or wheat germ lysate (Progema) was used to generate shifted and non-shifted protein products. In each assay, a total of 5 μl reaction containing 50–250 ng of capped reporter mRNA, 2.5 μl of translation lysate and 0.2 μl of 10 μCi/μl ^35^S-labeled methionine (NEN) was incubated at 30°C for 1.5–2 h. Samples were then resolved by 12% SDS-PAGE, and exposed to a phosphorimager screen for quantification after drying. Frameshifting efficiencies were calculated, by dividing the counts of the shifted product by the sum of the counts for both shifted and non-shifted products, with calibration of the methionine content in each protein product. All the radioactivity-based -1 PRF activity measurements *in vitro* were then performed by assuming that ribosome drop-off effect (15) was minimized for the translation of the shortened -1 frame product. As we present all of our *in vitro* -1 PRF results in term of relative -1 PRF activity, the ribosome drop-off effect was indeed removed by the procedure.

### Mammalian cell cultures, transfection and cell viability assay

Human embryonic kidney HEK-293T cells were cultured in Dulbecco's modified Eagle medium (Gibco) supplemented with 10% fetal bovine serum (FBS) (Gibco). One day before the transfection, 0.5–1 × 10^5^ HEK-293T cells per well were grown in a 24-well culture plate with 1 ml complete medium for 18 h. The medium was then replaced by 1% FBS of MEM for 1 h before the adding of the mixture of 0.5 μg plasmid DNA and jetPEI^TM^ transfection reagent (Polyplus) into each well according to the manufacturer's instructions. The 1% FBS-MEM containing theophylline (Sigma) or Adox (Sigma) was added 4 h after the transfection and cells were grown for 20 h before further analysis. To perform the MTT assay, thiazolyl blue tetrozolium Bromide (MTT, Sigma) was added to final concentration of 0.5 mg/ml and then incubated 3 h for crystal formation. The crystals were then dissolved by Dimethyl sulfoxide (DMSO) and measured for absorbance at 550 nm by enzyme-linked immunosorbent assay reader (TECAN). The results were then presented as cell viability by comparing with that of the non-treated 293T cells.

### Frameshifting activity analysis in 293T cells

Luciferase activity measurements for p2luc reporter transfected 293T cell lysates were performed using the Dual Luciferase^TM^ reporter assay (Promega) according to the manufacturer's instructions on a CHAMELEON^TM^ multi-label plate reader (HIDEX). Frameshifting efficiency was then calculated according to previously described procedure (15). For fluorescent imaging, the cells were washed with 1× PBS and observed by an epifluorscence microscope (Olympus BX51) and recorded with an Olympus DP71 camera system. The fluorescence filter setting (Olympus) and emission wavelengths used for Venus were U-MYFPHQ/550 nm. The transmitted light images were used to monitor the cell morphology.

### Western blotting

The transfected and ligand-treated cells were collected, lysed with Lysis buffer (50 mM HEPES-pH 7.5, 100 mM NaCl, 1 mM ethylenediaminetetraacetic acid, 0.5% triton X-100, 10% glycerol) and centrifuged to recover cell lysates. After protein concentration determination by Bradford assay (BioRad), 15 μg/well of total protein was loaded into a 12% SDS-PAGE electrophoresis for separation. The separated proteins were then transferred to a polyvinglidene difluoride (PVDF) membrane (PerkinElmer) by a Trans-Blot semidry blotting system (Bio-Rad). After blocking treatment, the membrane was treated with the primary antibody rabbit anti-GFP (polyclonal antibody, 1:1,000; BioVision) or mouse anti β-actin (monoclonal antibody, 1:5,000; Abcam), and followed by incubation with horseradish peroxidase-conjugated secondary antibody (anti-rabbit immunoglobulin G (IgG), 1:10,000; Jackson or anti-mouse IgG, 1:10,000; Jackson). The blot was then visualized by the addition of Western lighting plus ECL (PerkinElmer) and detected by a LAS-3000 luminescent image analyzer (FUJIFILM).

### Statistical analysis of experimental data

Experiments were performed in triplicate and the relative frameshifting activity was reported as one standard deviation from the mean.

## RESULTS

### Co-translational refolding of a -1 PRF attenuator hairpin and co-transcriptional folding of a prokaryotic transcriptional terminator hairpin

**Figure 1.**
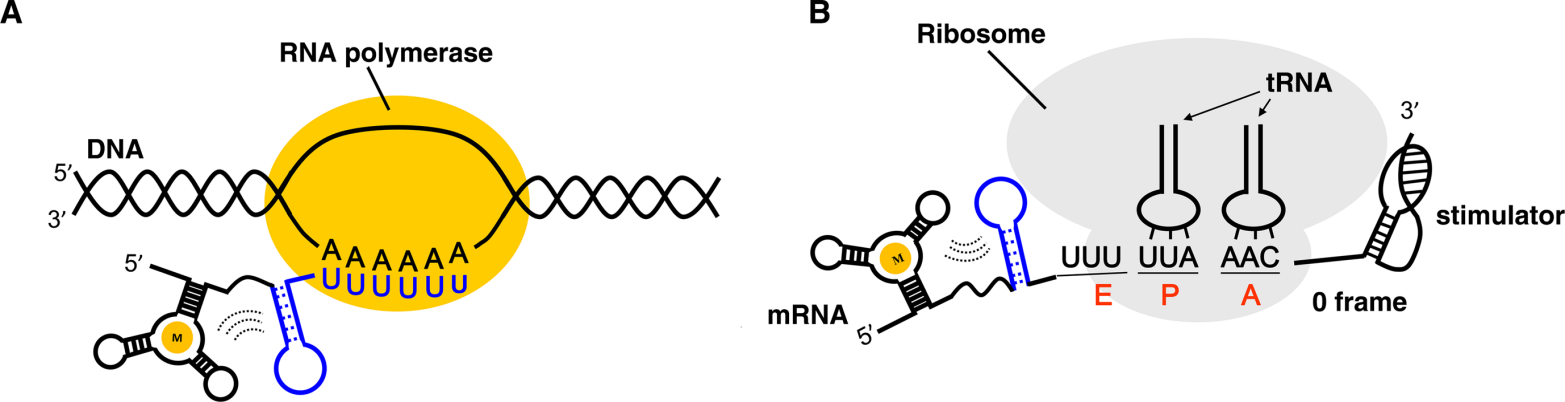
Comparison of co-transcriptional transcription terminator hairpin folding with co-translational -1 PRF attenuator hairpin refolding. (**A**) Prokaryotic transcriptional termination can be triggered by a folding GC-rich terminator hairpin with a downstream U-stretch sequence in the transcribed RNA, and be further regulated by a ligand-sensing element upstream of the terminator hairpin via the ligand-induced RNA conformation rearrangement. (**B**) Scheme showing that a stable hairpin upstream of the slippery site (UUUAAAC) and a downstream pseudo-knot stimulator possess opposite effects on -1 PRF activity, while the upstream hairpin formation might be regulated by a nearby ligand-sensing RNA element.

We have previously shown that a hairpin upstream of the -1 PRF slippery site can attenuate -1 PRF efficiency (14). In this case, attenuation efficiency is determined by hairpin stability and its distance from the slippery site (14). We noted that this co-translational refolding RNA hairpin (Figure 1) was reminiscent of co-transcriptional folding RNA hairpins that modulate the ρ-independent transcriptional termination efficiency in prokaryotic systems (18) (Figure 1), and reasoned that this -1 PRF attenuator hairpin might be regulated in ways similar to hairpins in the prokaryotic transcription termination (18). This means ligand-dependent regulation of the upstream attenuator hairpin formation provides an alternative way in building ligand-responsive -1 PRF regulatory circuits in addition to using a downstream ligand-responsive pseudo-knot stimulator (10,11).

### Regulation of -1 PRF by modulating co-translational refolding of an attenuator hairpin through RNA-protein interactions

**Figure 2.**
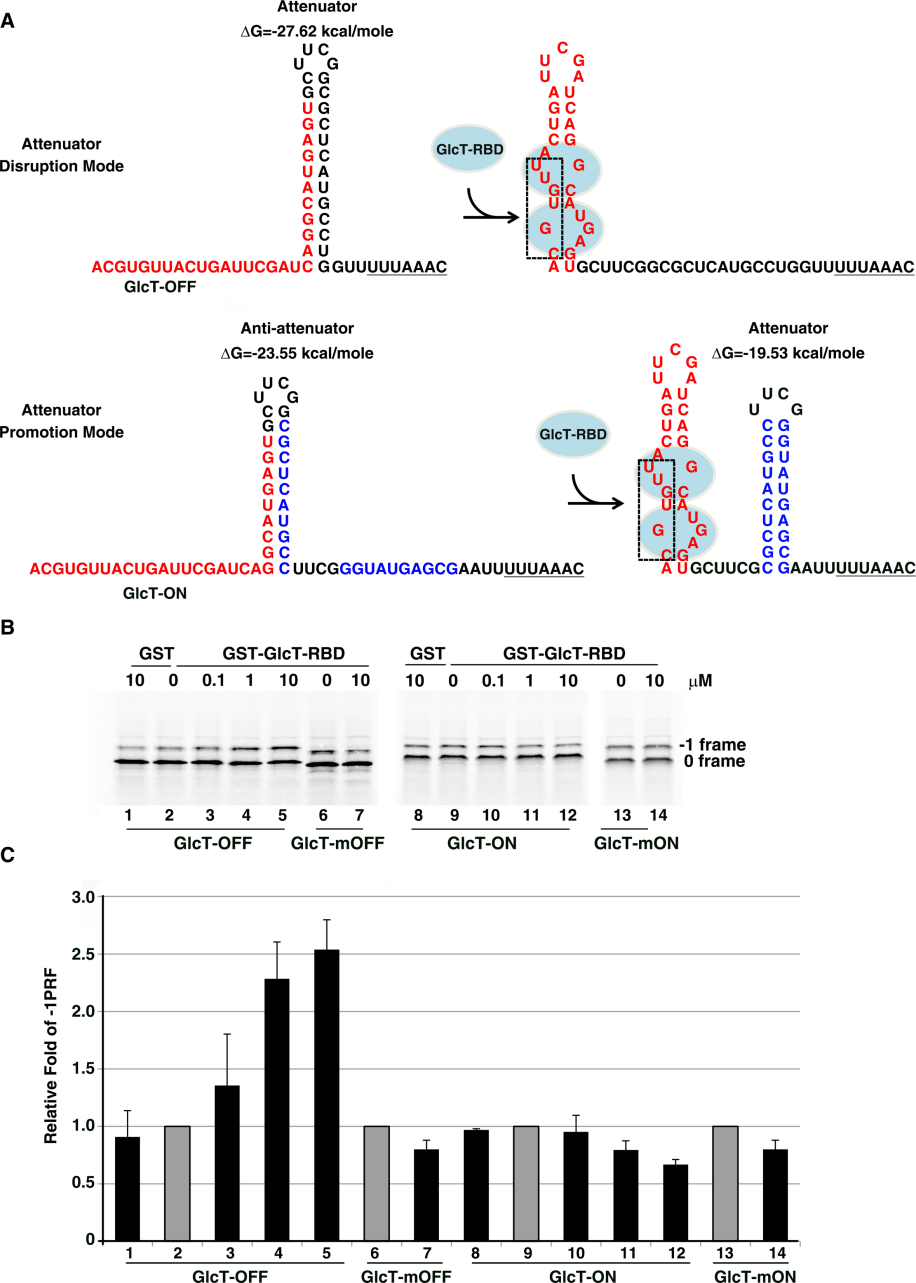
GlcTRBD-dependent regulation of -1 PRF in reticulocyte lysate. (**A**) Sequence designs for RNA-protein interactions mediated -1 PRF attenuator hairpin disruption and promotion in GlcT-OFF and GlcT-ON RNA elements, respectively. Mfold (20) was used to predict structures and free energies of the attenuator and anti-attenuator hairpins. The RNA sequences crucial in GlcT-binding were deleted (boxed) in control elements, GlcT-mOFF and GlcT-mON. (**B**) *In vitro* radioactivity-based -1 PRF activity analysis using a shortened p2luc reporter in the presence of increasing amounts of GlcTRBD. The -1 PRF modules contain a potent downstream DU177 pseudo-knot stimulator in combination with different upstream chimeric elements as shown in (A). (**C**) The relative -1 PRF activity of reporter constructs in (A) in the presence of different dosages of GlcTRBD with the GlcTRBD-free activity being treated as 1 (in gray) based on the results from (B).

To test this hypothesis, we used the well-documented RNA-protein interactions of transcriptional antiterminator system in *B. subtilis* (19) to see if -1 PRF could be regulated in-trans by an RNA-protein recognition module engineered to affect upstream -1 PRF attenuator hairpin formation. As the RAT sequence of *B. subtilis ptsGHI* operon adopts an unstable internal loop conformation and can be stabilized by the binding of GlcT antiterminator protein (Supplementary Figure S1) (19), we designed a chimeric RNA element containing an RAT sequence with the 3′-side of RAT internal loop embedded in a stable hairpin stem predicted by Mfold (20). (Figure 2A) and placed the chimeric RNA element upstream of the slippery site with a potent downstream -1 PRF stimulator, the DU177 pseudo-knot (21). We rationalized that the engineered hairpin is the dominant conformation and serves as an upstream -1 PRF attenuator, whereas the addition of GlcT protein should disrupt the attenuator hairpin via RAT-GlcT complex formation and thus restore -1 PRF activity. In this context, we reasoned that the elongating ribosome would help unwind the attenuator hairpin stem that might trap the 3′-side of RAT element and facilitate transient RAT-GlcT interaction. Additionally, a recent ribosome profiling study revealed the lack of packed ribosomes at the ribosomal pause sites of genes expressed in mouse embryonic stem, suggesting low encounter frequency between elongating ribosomes (22). Therefore, the designed protein-mediated RNA conformation rearrangement could exist to affect a downstream frameshifting ribosome before the arrival of an upstream ribosome.

The -1 PRF efficiency of a reporter containing the designed upstream chimeric element (GlcT-OFF) was up-regulated upon addition of the purified GlcT protein in a dosage-dependent manner (compare lanes 2–5 in Figure 2B and C) based on *in vitro* frameshifting assay performed in reticulocyte lysate. We then designed the second chimera to down-regulate the -1 PRF by the formation of an efficient attenuator hairpin promoted by the same RNA-protein interactions (Figure 2A). The 5′ part of attenuation hairpin and 3′ part of RAT internal loop were adjusted to form a stable hairpin (the anti-attenuator) that does not act as a -1 PRF attenuator due to its distant spacing from the slippery site (14). Therefore, the GlcT-ON RNA element is embedded with three potential RNA motifs; that is, an RAT internal loop, an anti-attenuator hairpin and an efficient -1 PRF attenuator hairpin with the component of the anti-attenuator hairpin being overlapped with the other two motifs. In principle, this anti-attenuator hairpin might be the dominant conformation without the GlcT protein. However, the equilibrium could be driven by RNA-protein interaction to disrupt the anti-attenuator hairpin stem upon GlcT protein addition and thus release the 3′-side of anti-attenuator hairpin to facilitate the formation of an efficient -1 PRF attenuator hairpin. Consistently, the *in vitro* -1 PRF activity of a reporter containing the second chimeric element (GlcT-ON) was repressed by the purified GlcT protein in a dosage-dependent manner (compare lanes 9–12 in Figure 2B and C) although the degree of response to GlcT addition was not as significant as that in GlcT-OFF. The relatively weak -1 PRF activity in GlcT-free GlcT-ON compared with that of GlcT-OFF in the presence of 10 μM GlcT (compare lane 5 with lane 9 in Figure 2B) implicates that the compromised dynamic range may be caused by partial formation of a functional attenuator hairpin in the GlcT-free GlcT-ON. Finally, mutants (GlcT-mOFF and GlcT-mON) with the GlcT-binding pocket deleted in the RAT element of GlcT-ON and GlcT-OFF did not respond to the addition of 10 μM of GlcT protein significantly (compare lanes 6 with 7 as well as lanes 13 with 14 in Figure 2B and C), suggesting the regulation is mediated by specific GlcT-RAT interaction. Taken together, these proof-of-principle experiments indicate that -1 PRF activity can be regulated in-trans via a set of RNA-protein interactions designed to modulate the formation of an upstream -1 PRF attenuation hairpin.

### RNA-protein interactions can be replaced by RNA-small molecule interactions for attenuator hairpin regulation

**Figure 3.**
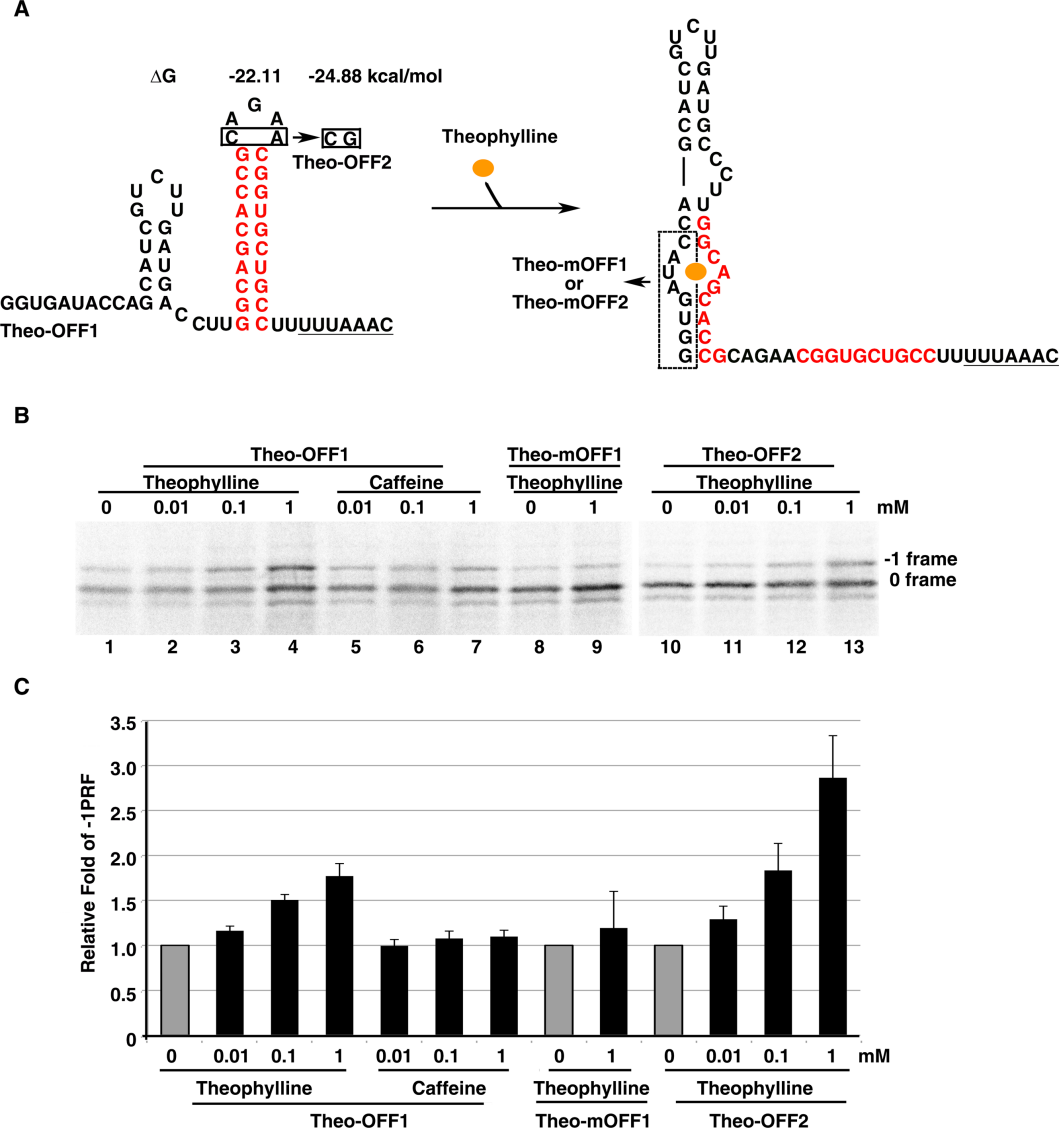
Dynamic range of theophylline-dependent regulation of -1 PRF in reticulocyte lysate is improved by stabilizing the attenuator hairpin. (**A**) The sequences, predicted structures and free energies of theo-OFF1, theo-mOFF1, theo-OFF2 and theo-mOFF2. The boxed nucleotides in theophylline-binding pocket were deleted in the negative control theo-mOFF1 and theo-mOFF2 elements. (**B**) *In vitro* radioactivity-based -1 PRF activity analysis of a potent downstream DU177 pseudo-knot stimulator with an upstream theo-OFF1/2 or theo-mOFF1/2 elements using a shortened p2luc reporter in the presence of increasing amounts of theophylline or caffeine. (**C**) The relative -1 PRF activity of reporter constructs in (B) in the presence of different dosages of theophylline or caffeine with the ligand-free activity being treated as 1 (in gray).

**Figure 4.**
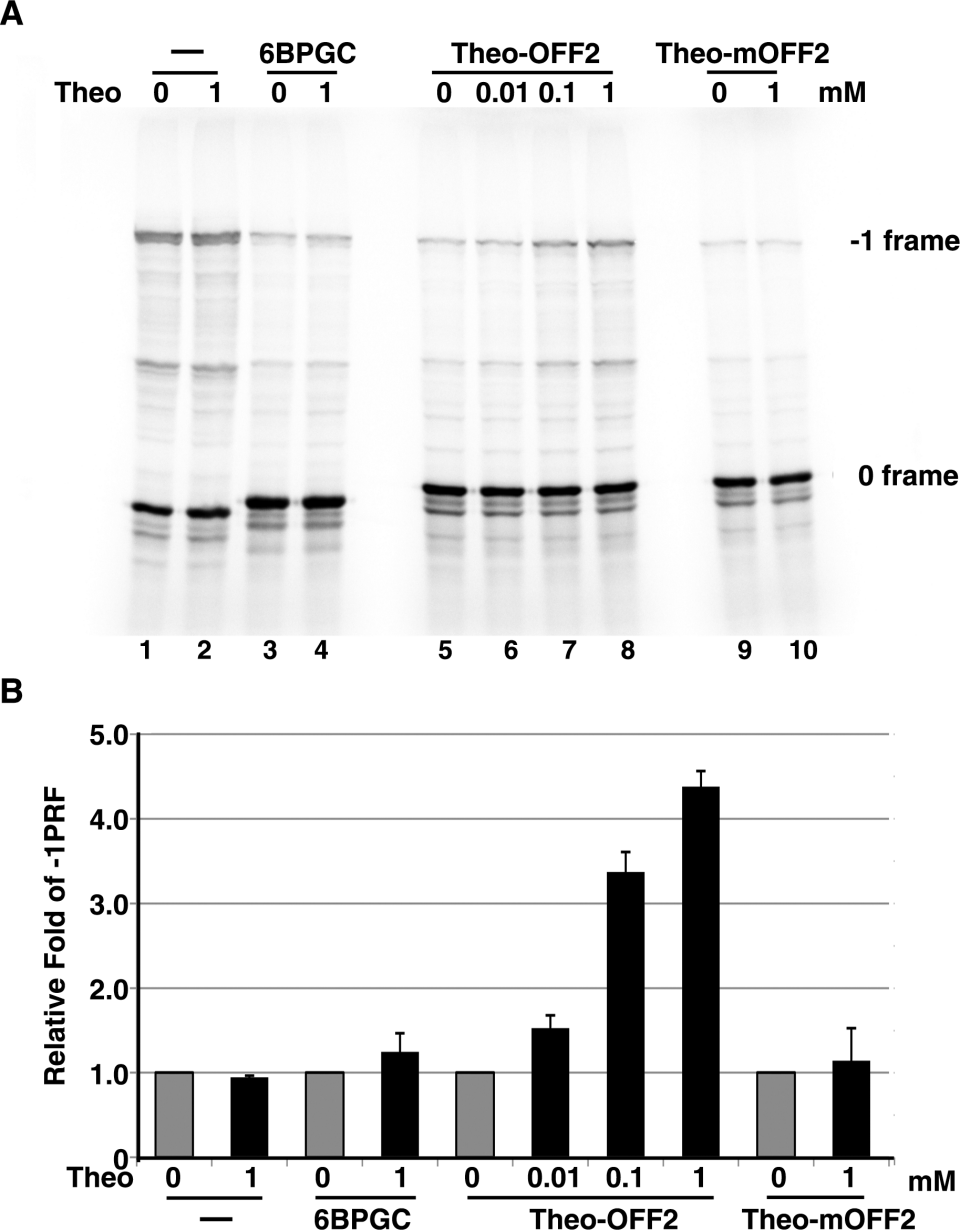
The dynamic range of theophylline-dependent regulation of -1 PRF by theo-OFF2 enhanced further in wheat germ lysate. (**A**) Radioactivity-based -1 PRF activity analysis of a potent downstream DU177 pseudo-knot stimulator with an upstream theo-OFF2 or control element using p2luc reporter in the presence of increasing amounts of theophylline with the wheat germ extract being used as the *in vitro* translation system. 6BPGC is a potent upstream attenuator lacking a theophylline aptamer as described previously (14). (**B**) Relative fold change in -1 PRF activity for reporter constructs in (A) in the presence of different dosages of theophylline compared with the activity of constructs without theophylline addition (in gray).

To facilitate the application of regulatory -1 PRF attenuation hairpins in cells and to extend the regulatory module to small ligand-RNA interactions, we explored the possibility of replacing the RAT-GlcT interaction with the theophylline aptamer-theophylline interaction (23), given the cell-permeable property of theophylline (Supplementary Table S1) (24). To this end, a designed -1 PRF attenuation hairpin was constructed with its 5′-stem sequences being complementary to the 3′-side sequences of a high-affinity theophylline aptamer (23) (Figure 3A). Similar to RAT-GlcT-dependent regulation, the *in vitro* -1 PRF activity of a DU177 pseudo-knot stimulator driven reporter, harboring this designed upstream theo-OFF1 element, was up-regulated upon theophylline addition in a dosage-dependent manner, whereas mutant with the theophylline-binding pocket deleted (theo-mOFF1) did not respond to theophylline (Figure 3B and C). Given the less than 1-fold of -1 PRF regulation by theo-OFF1, we reasoned that the substantial stability in theophylline aptamer could reduce the population of the attenuator hairpin to impair its attenuation efficiency and the resultant dynamic range. We then modified the sequence in the attenuation hairpin loop of theo-OFF1 element without changing the theophylline-RNA interaction, and found that the dynamic range could be improved further by stabilizing the attenuator hairpin element (theo-OFF2) (Figure 3B and C). Interestingly, an even higher dynamic range of theophylline-dependent -1 PRF activity was observed in wheat germ lysate (Figure 4) suggesting this theophylline-dependent -1 PRF regulatory module may also work in plants.

### Regulation of -1 PRF by RNA-small molecule interactions in mammalian cells

**Figure 5.**
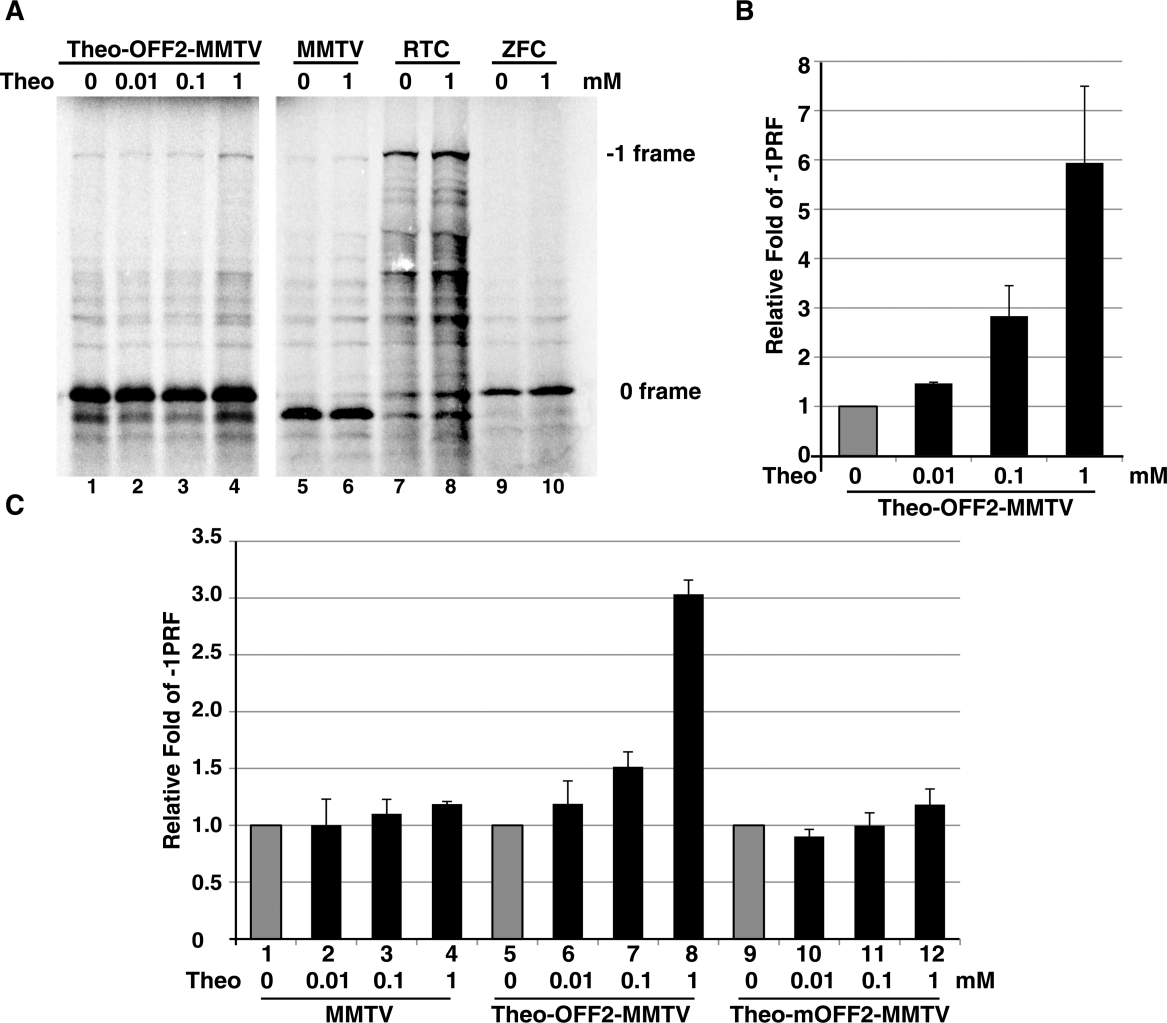
The theophylline-dependent regulation mediated by theo-OFF2 element can be applied to control -1 PRF activity driven by an MMTV pseudo-knot. (**A**) *In vitro* radioactivity-based -1 PRF activity analysis of MMTV pseudo-knot-based constructs in Supplementary Figure S2 using full-length p2luc reporter in the presence of increasing amounts of theophylline or caffeine. (**B**) The relative -1 PRF activity of Theo-OFF2-MMTV related reporter constructs in (A) in the presence of different dosages of theophylline or caffeine with ligand-free activity being treated as 1 (in gray). (**C**) The relative -1 PRF activity of Theo-OFF2-MMTV related reporter constructs in 293T cell in the presence of different dosages of theophylline or caffeine with the ligand-free activity being treated as 1 (in gray). The relative -1 PRF activity was calculated by calibrating with the dual-luciferase activity of RTC in Supplementary Figure S2 according to the published procedures (15).

**Figure 6.**
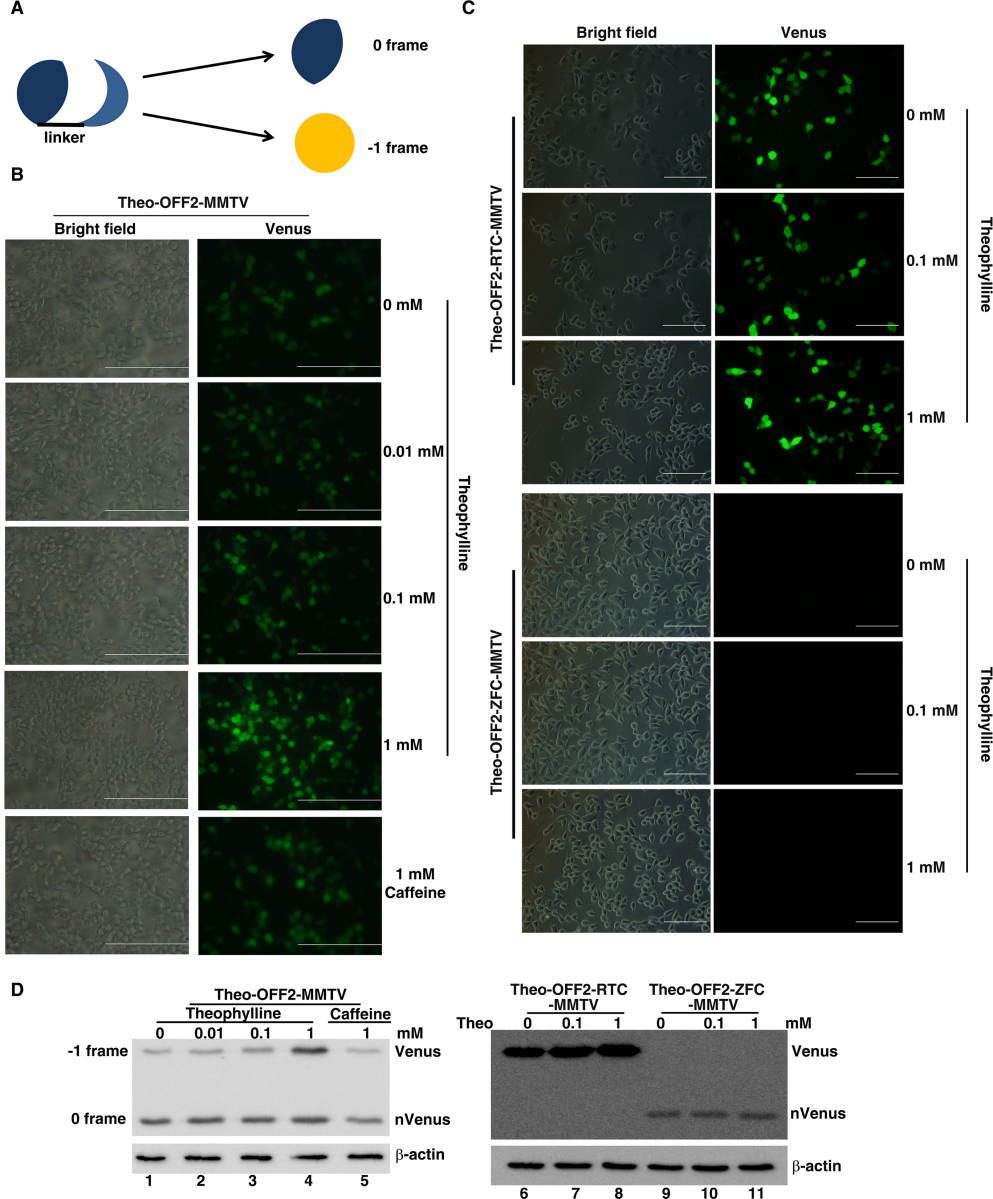
A fluorescence-based analysis of theophylline-dependent regulatory switch of -1 PRF in 293T cells. (**A**) Scheme of the reconstitution of split Venus by a linker (composed of ligand-responsive -1 PRF elements) to monitor -1 PRF activity in 293T cell. (**B**) Fluorescence microscopy images of 293T cells, transfected with a pNinsertC-Venus -1 PRF reporter (Theo-OFF2-MMTV) harboring a Theo-OFF2 element and an MMTV pseudo-knot, in the presence of different amounts of theophylline. (Scale bar, 10 μm.) (**C**) Fluorescence microscopy images of 293T cells transfected with the RTC and ZFC of Theo-OFF2-MMTV in the presence of different amounts of theophylline. (Scale bar, 10 μm.) (**D**) Western blot results of 293T cell lysates from cells transfected with the -1 PRF reporters in (B) and (C). The N-Venus (corresponding to 0 frame product) and the C-Venus containing full-length product (corresponding to -1 frame product) were detected by a polyclonal anti-GFP antibody. The cellular β-actin was treated as the internal loading control.

To see if the ligand-responsive -1 PRF regulatory element upstream of the slippery site function in other downstream stimulator pseudo-knots, we replaced the DU177 pseudo-knot with a minimal MMTV pseudo-knot (25) (Supplementary Figure S2A) and measured the theophylline-dependent -1 PRF activity using a full-length p2luc reporter in reticulocyte lysate as well as 293T cell. We found that the theo-OFF2-MMTV construct also regulated the -1 PRF stimulated by a downstream MMTV pseudo-knot in a theophylline-dependent way, whereas constructs with abolished frameshifting elements (RTC and ZFC in Supplementary Figure S2B) or with impaired theophylline-binding elements (MMTV and theo-mOFF2-MMTV) did not respond significantly to theophylline variation (Figure 5). These results suggest the general application of upstream theophylline-responsive element in regulation of the -1 PRF stimulated by different downstream stimulators. To further demonstrate the application in mammalian cells, the theophylline-dependent -1 PRF regulatory element was then evaluated in 293T cell using a fluorescent reporter. As separated N- and C-terminal domains of YFP work in-trans to reconstitute a functional fluorescent protein (26), we used the -1 PRF switch to link the split N and C domains of Venus (derived from YFP) with the coding region of C Venus being shifted to the -1 frame (Figure 6A). Accordingly, in this approach, Venus activity observation would be an indication of -1 frameshifting. We found that prominent Venus activity could be induced by theophylline but not by caffeine (Figure 6B and D). By contrast, control constructs (RTC and ZFC) did not respond to theophylline variation (Figure 6C and D). Collectively, these experiments demonstrate that the dynamics of a co-translational refolding hairpin can be controlled to achieve reading-frame switch regulation in different eukaryotic systems.

### Synergetic regulation of -1 PRF activity by combining ligand-responsive stimulator and attenuator

**Figure 7.**
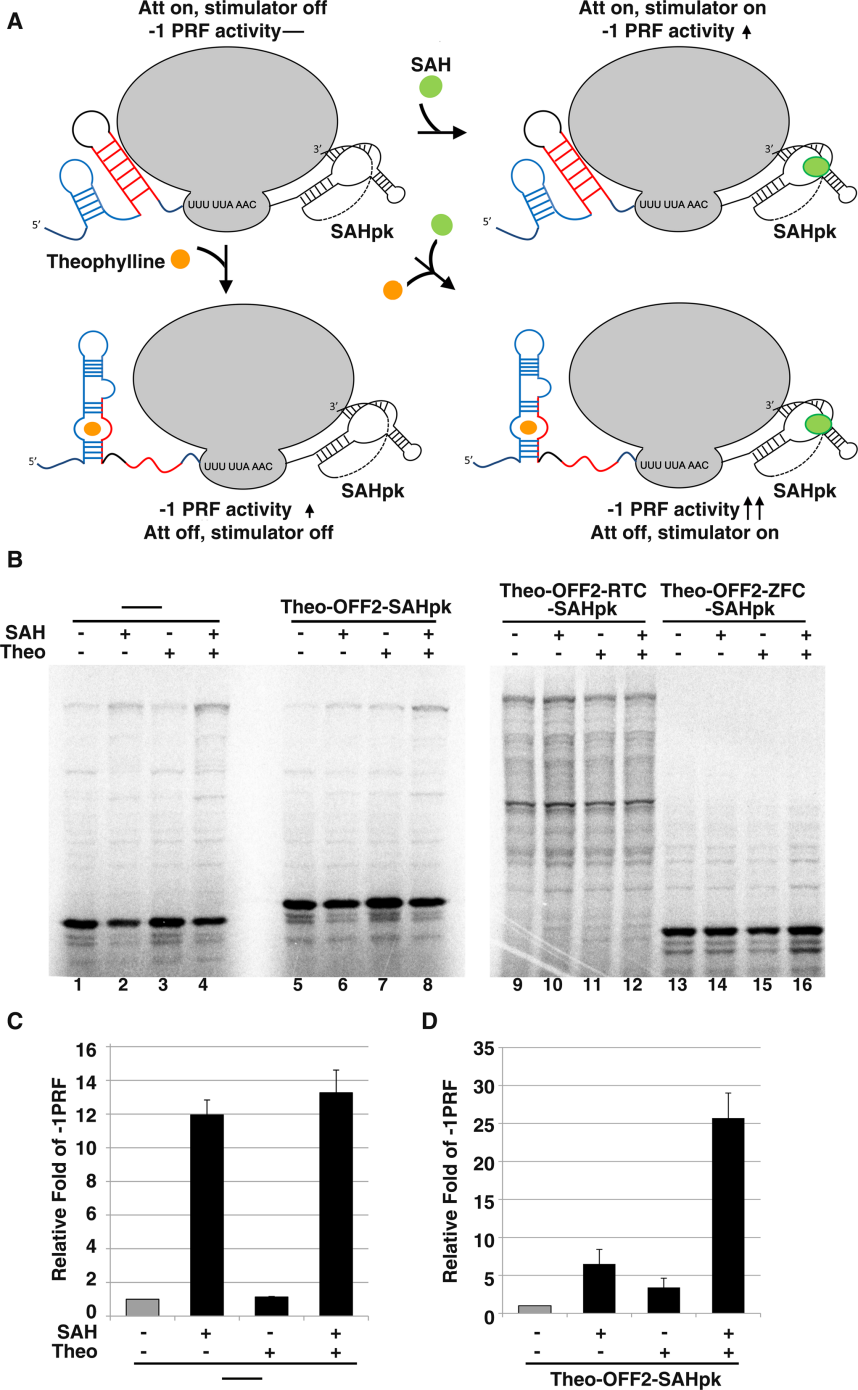
Synergetic regulation of -1 PRF by SAH and theophylline. (**A**) Scheme of a two-input logic gate built by combining an SAH-dependent stimulator with an upstream theophylline-dependent attenuator of -1 PRF. (**B**) Radioactivity-based -1 PRF activity analysis using p2luc reporter in wheat germ extract. The -1 PRF activity of reporters containing an SAH-sensing pseudo-knot stimulator (10) with (lanes 5–8) or without (lanes 1–4) an upstream theo-OFF2 as well as with related control elements (lanes 9–16 for RTC and ZFC similar to those in Supplementary Figure S2) were measured as indicated. The concentration used for theophylline and SAH were 1 mM and 100 μM, respectively. (**C**) Relative fold change in -1 PRF activity for reporter construct lacking the theo-OFF2 element in (B) in the presence of different ligands, compared with the activity of constructs without ligand addition (in gray). (**D**) Relative fold change in -1 PRF activity for reporter construct carrying the theo-OFF2 element in (B) in the presence of different ligands, compared with the activity of constructs without ligand addition (in gray).

We have demonstrated that -1 PRF could be stimulated by a metabolite, *S*-adenosylhomocysteine (SAH) via an SAH-responsive pseudo-knot stimulator (10). The ability to regulate -1 PRF in opposite directions by small molecule-induced conformational rearrangements of the attenuator and stimulator provides a unique opportunity to build an ingenious ligand-dependent regulatory circuit using the -1 PRF as platform. With potential application in both animals and plants for synergetic -1 PRF control by different lignds (Figure 7A), we then fused theo-OFF2 with an SAH-dependent -1 PRF stimulator (10) and examined the ligand-dependent -1 PRF activity *in vitro* using wheat germ lysate. The -1 PRF activity was close to the background level without the addition of theophylline or SAH, while the addition of theophylline or SAH only mildly increased -1 frameshifting efficiency (compare lanes 5–7 in Figure 7B and D). However, the addition of both theophylline and SAH enhanced -1 frameshifting in a synergetic way (lane 8 in Figure 7B and D). By contrast, a reporter containing only the SAH-dependent -1 PRF stimulator did not respond significantly to theophylline alone (compare lane 1 with lane 3 as well as lane 2 with lane 4 in Figure 7B and C), while frameshifting-impaired control reporters (RTC and ZFC) did not respond to either ligands (lanes 9–16 in Figure 7B). Thus, combining two ligand-responsive -1 PRF regulators further enhances the dynamic range in regulation of -1 PRF activity.

**Figure 8.**
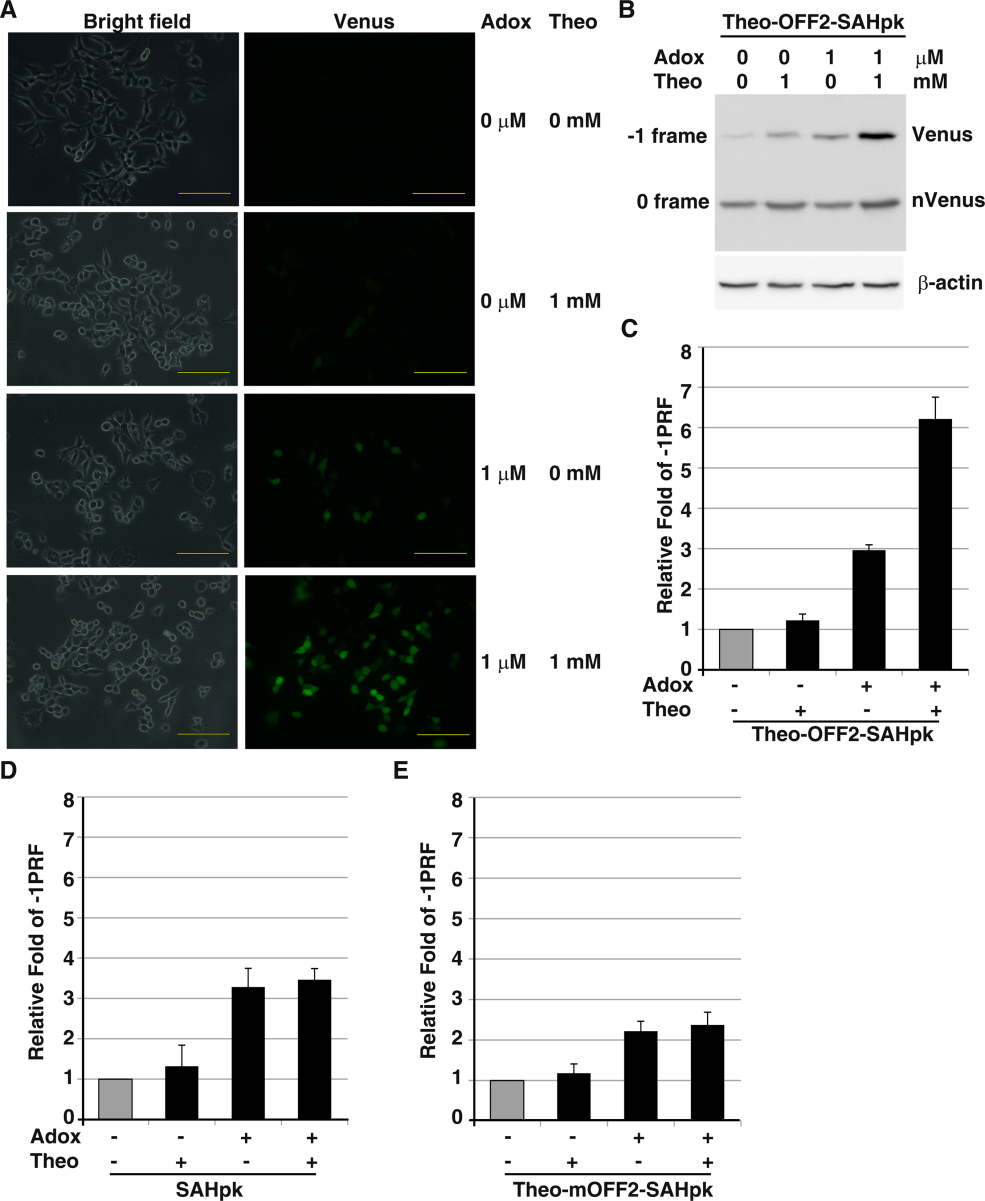
A potential two-input logic gate built upon ligand-dependent regulation of -1 PRF stimulation and attenuation. (**A**) Fluorescence microscopy images of 293T cells, transfected with a pNinsertC-Venus -1 PRF reporter harboring an SAH-responsive pseudo-knot and the upstream theo-OFF2 element, in the presence of different amounts of theophylline and Adox. (Scale bar, 10 μm.) (**B**) Western blot results of 293T cell lysates from cells transfected with the -1 PRF vector in (A). The N-Venus (corresponding to 0 frame product) and the C-Venus containing full-length product (corresponding to -1 frame product) were detected by a polyclonal anti-GFP antibody. The cellular β-actin was treated as the internal loading control. (**C**) Relative fold change in -1 PRF activity of SAH-PK containing p2luc reporter construct carrying the upstream theo-OFF2 element in the presence of different ligands, compared with the activity of construct without ligand addition (in gray) in 293T cells using the same condition as in (A). (**D**) Relative fold change in -1 PRF activity of SAH-PK containing p2luc reporter construct lacking the theo-OFF2 element in 293T cells using the same condition as in (A). (**E**) Relative fold change in -1 PRF activity of SAH-PK containing p2luc reporter construct carrying the theo-mOFF2 element in 293T cells using the same condition as in (A).

This potential two-input regulatory -1 PRF switch was then evaluated in 293T cell using the Venus-based fluorescent reporter. Aadenosine- 2′, 3′ dialdehyde (Adox) (Supplementary Table S1), a cell-permeable AdoHcy hydrolase inhibitor that blocks SAH hydrolysis to enhance intracellular SAH concentration (27), was used to adjust cellular SAH concentration. We found that prominent Venus activity could be observed in the presence of both theophylline and Adox (Figure 8A and B), whereas control reporters lacking the theo-OFF2 element responded to Adox only (Supplementary Figure S3). By contrast, the -1 PRF activity of constructs with impaired frameshifting mutation did not respond to either ligand (Supplementary Figure S3). Consistently, results from quantitative measurement of ligand-dependent -1 PRF activity in 293T cell using p2luc reporter (Figure 8C–E) indicated similar trends as those observed in wheat germ lysate (Figure 7). Furthermore, no cellular toxicity was observed for the ligand-treated 293T cell within the regulatory dosages based on cell viability assay (Supplementary Figure S4). Together, these experiments clearly demonstrate the potential of -1 PRF activity regulation in building a two-input logic gate by using small ligands that modulate the RNA conformations of two RNA motifs of opposite role in -1 PRF.

## DISCUSSION

Here, we present the concept, design and application of ligand-responsive RNA elements in the regulation of -1 PRF activity that enable the investigators to explore the integration of metabolite-riboswitch interactions into translational regulation in mammalian cells. Strictly speaking, the potential involvement of ribosome in RNA rearrangement processes makes this system not fit to the definition of a riboswitch (2,3) and be more similar to *trp* operon attenuation (28). Notably, we demonstrate the ligand-dependent regulatory potential of a translational refolding RNA hairpin in ribosomal reading-frame switch control, side-stepping the need to look for a ligand-responsive pseudo-knot. Given the observation of -1 PRF-dependent nonsense-mediated decay (NMD) in the tuning of immune responses of mammalian systems (29), this regulatory -1 PRF approach may provide a mean to deliver ligand-dependent regulation of NMD to tune the expression level of specific genes of interest. Additionally, it provides a unique way to regulate -1 PRF in a synergetic manner while being combined with a downstream ligand-responsive stimulator.

However, the optimal size limit of an upstream regulatory RNA switch needs further investigations because the function of a large regulatory RNA element could be compromised by the spacing between two elongating ribosomes during translation (21,30). Interestingly, the distinct dynamic range to ligand-response observed between reticulocyte lysate and wheat germ extract suggests the existence of system-dependent factors and is worth of further analysis. The improvement of regulatory dynamic-range, by rational redesign of attenuator hairpin and replacing a potent stimulator with a weaker stimulator, suggests that this platform will benefit further from deeper understanding of the programmed reading-frame switch mechanism. Particularly, the weak but significant GlcT-dependent -1 PRF repression observed in GlcT-ON could be improved further with more mechanism insight of a functional attenuator hairpin. Given the numerous RNA-responsive ligands provided by ample examples within prokaryotic transcription and translation regulation (2–3,18) as well as the development of *in vitro* selection tools (31), we expect that our findings should make the use of ligand-responsive RNA devices in translational regulation of eukaryotic and mammalian cells more accessible for creating new gene-regulatory circuits in synthetic biology (6,7).

## SUPPLEMENTARY DATA

Supplementary Data are available at NAR Online.

SUPPLEMENTARY DATA
