# Supplementary material for: Synergetic regulation of translational reading-frame switch by ligand-responsive RNAs in mammalian cells
Source: Nucleic Acids Res. 2014 Nov 20;42(22):14070–82. doi: 10.1093/nar/gku1233 (PMC4267651; doi:10.1093/nar/gku1233)
Supplement: SUPPLEMENTARY DATA [file supp_42_22_14070__index.html]

Synergetic regulation of translational reading-frame switch by ligand-responsive RNAs in mammalian cells — Synergetic regulation of translational reading-frame switch by ligand-responsive RNAs in mammalian cells — SUPPLEMENTARY DATA 

# Synergetic regulation of translational reading-frame switch by ligand-responsive RNAs in mammalian cells

## SUPPLEMENTARY DATA

**Files in this Data Supplement:**

- SUPPLEMENTARY DATA
