# Supplementary material for: Synergetic regulation of translational reading-frame switch by ligand-responsive RNAs in mammalian cells
Source: Nucleic Acids Res. 2014 Nov 20;42(22):14070–82. doi: 10.1093/nar/gku1233 (PMC4267651; doi:10.1093/nar/gku1233)
Supplement: SUPPLEMENTARY DATA [file supp_gku1233_nar-02937-r-2014-File003.pdf]

**Supplementary Information**

**"Synergetic regulation of translational reading-frame switch by ligand-responsive RNAs in mammalian cells"**

by Hsiu-Ting Hsu[‡], Ya-Hui Lin[‡], and Kung-Yao Chang[★]

Institute of Biochemistry, National Chung-Hsing University, 250 Kuo-Kung Road, Taichung, 402 Taiwan

**This supplement includes 4 supplementary figures with 1 supplementary table, and is organized in the following order:**

1. Supplementary Figures and Legends.
2. Supplementary Table and Legend.
3. Supplementary references.

**Supplementary Figures and Legends.**

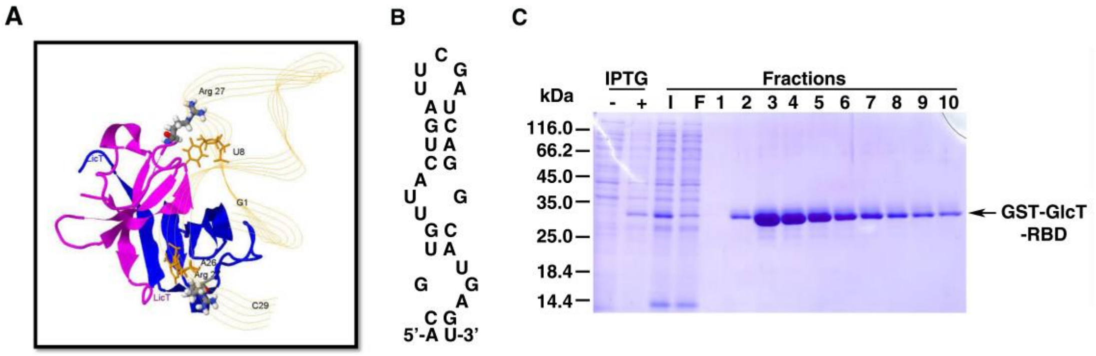


**Supplementary Figure 1.** Illustration of RNA-protein interactions in a transcriptional antiterminator complex of *Bacillus subtilis*. (**A**) Solution structure of LicT-RNA antiterminator complex (1) displayed via Jmol (2) based on the coordinates provided in PDB 1L1C. (**B**) The RAT sequences for GlcT antiterminator protein recognition that used in this study. (**C**) SDS-PAGE purification result of GST-GlcTRBD fusion protein.

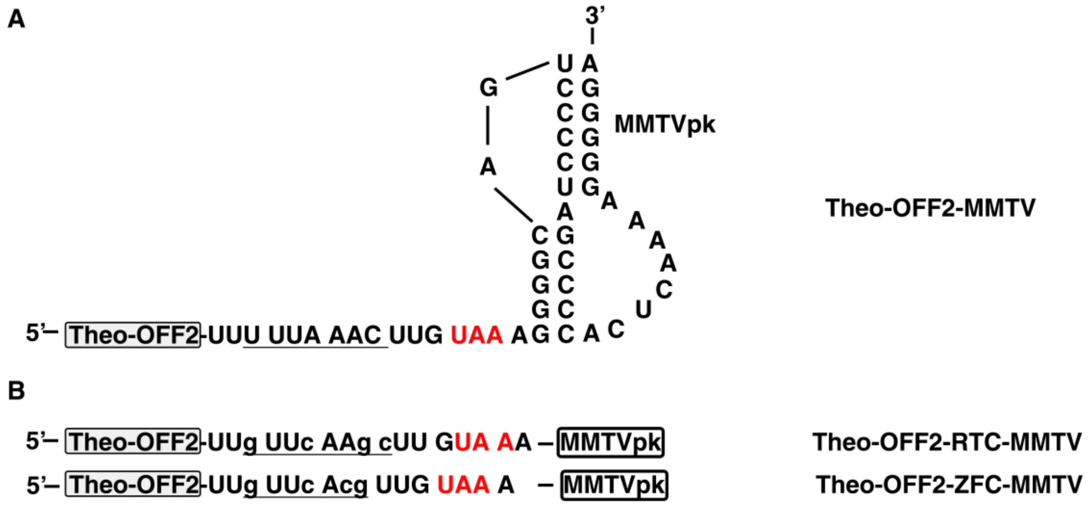


**Supplementary Figure 2.** The sequence of MMTV pseudoknot and related control elements. (**A**) The sequences and predicted secondary structures of the minimal MMTV -1 PRF pseudoknot stimulator with an upstream theo-OFF2 element used in theophylline-dependent -1 PRF activity analysis. (**B**) The sequences of read-through control (RTC) and zero-frame control (ZFC). The RTC will lead to formation of a full-length translation product without frameshifting via mutations in slippery site (underlined) to impair both -1 PRF and 0-frame termination (out of frame after mutation), whereas the ZFC will lead to formation of a shortened translation product without frameshifting due to mutations in slippery site (underlined) to impair -1 PRF with the 0-frame stop codon remaining in-frame. The UAA stop codons within different reading-frames of the constructs in (A) and (B) were typed in red, while the mutated nucleotides in the slippery sites were typed in lower case.

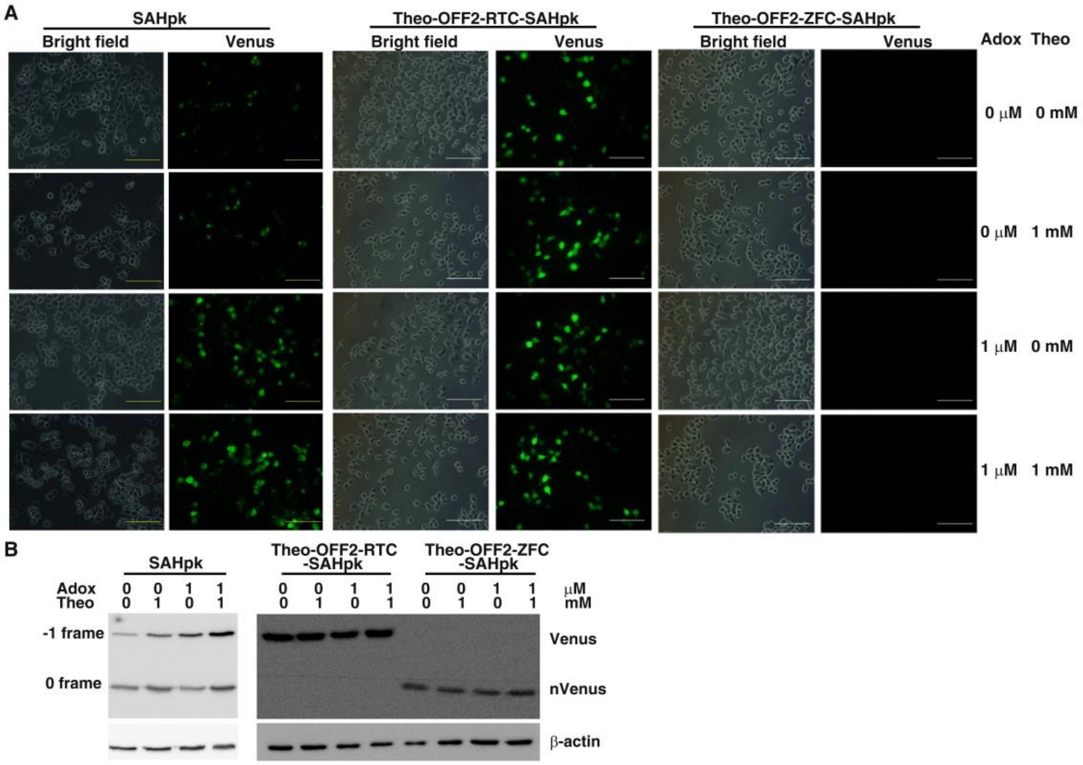


**Supplementary Figure 3.** The control elements with frameshifting impaired mutation in the slippery site of theo-OFF2-SAHPK element do not respond to Adox and theophylline variation in 293T cells. (**A**) Fluorescence microscopy images of 293T cells, transfected with a pNinsertC-Venus -1 PRF reporter harboring the theo-OFF2-SAHPK related control elements in the presence of different amounts of theophylline and Adox. (Scale bar, 10 μm) The compositions of RTC and ZFC were the same as those in supplementary Fig. 2B except that different stimulator pseudoknots were used, while the SAHPK construct lacked the upstream theo-OFF2 element. (**B**) Western blot results of 293T cell lysates from cells transfected with the -1 PRF reporters in (A). The N-Venus (corresponding to 0 frame product) and the C-Venus containing full-length product (corresponding to -1 frame product) were detected by a polyclonal anti-GFP antibody. The cellular β-actin was treated as the internal loading control.

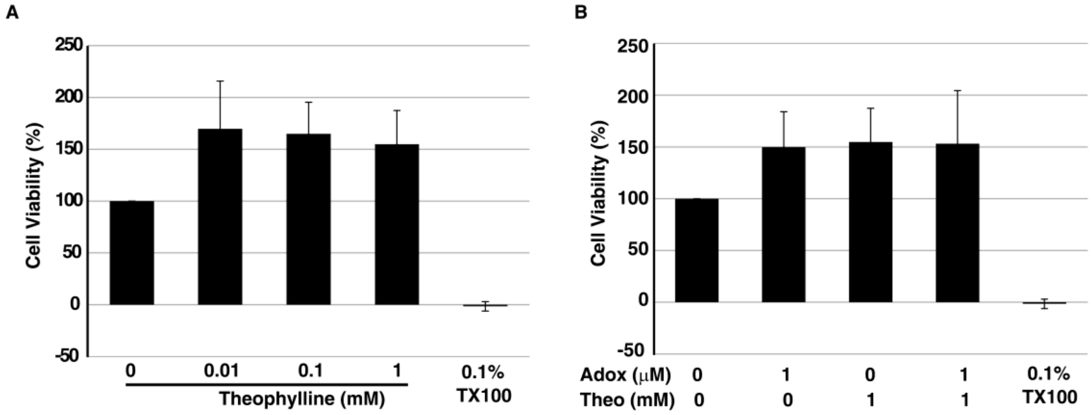


**Supplementary Figure 4.** The effect of Adox and Theophylline on cell viability. (**A**) Cell viability of 293T cell upon theophylline treatment was based on MTT assay results using that of ligand-free cell as 100%, while that of cells treated with 0.1% Triton X-100 was used as negative control. (**B**) Cell viability of 293T cell in the presence of different amounts of theophylline and Adox based on MTT assay results using that of non-treated cells as 100%.

**Supplementary Table and Legend.**

| Name | Structure |
|---|---|
| **Caffeine** | 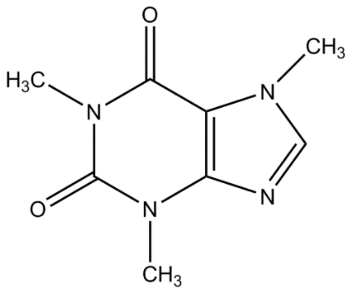 |
| **Theophylline** | 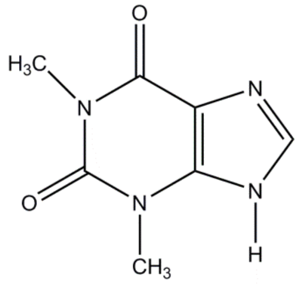 |
| **S-adenosylhomocysteine (SAH)** | 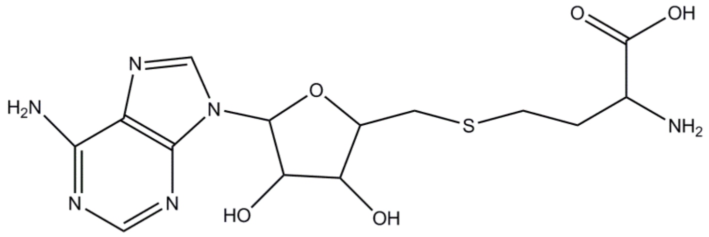 |
| **Adenosine-2′,3′-dialdehyde (Adox)** | 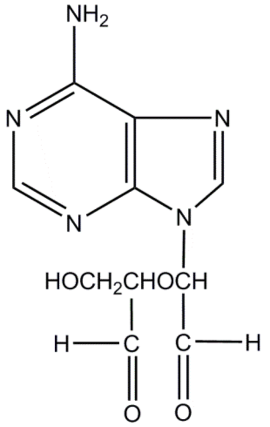 |

**Supplementary Table 1.** Chemical structures of small molecule ligand used. The four small molecule reagents used in this study were all purchased from Sigma.

**Supplementary References**


1. Yang, Y., Declerck, N., Manival, X., Aymerich, S., & Kochoyan, M. Solution structure of the LicT-RNA antitermination complex: CAT clamping RAT. *EMBO. J.* **21**, 1987-1997 (2002).
2. Hanson, R. M. *Jmol*– a paradigm shift in crystallographic visualization. *J. Appl. Cryst.* **43**, 1250-1260 (2010).
